# Serum glial fibrillary acidic protein and neurofilament light chain in patients with early treated phenylketonuria

**DOI:** 10.3389/fneur.2022.1011470

**Published:** 2022-09-29

**Authors:** Amelie S. Lotz-Havla, Sabrina Katzdobler, Brigitte Nuscher, Katharina Weiß, Johannes Levin, Joachim Havla, Esther M. Maier

**Affiliations:** ^1^Department of Pediatrics, Dr. von Hauner Children's Hospital, University Hospital, LMU Munich, Munich, Germany; ^2^Department of Neurology, University Hospital, LMU Munich, Munich, Germany; ^3^German Center for Neurodegenerative Diseases, site Munich, Munich, Germany; ^4^Munich Cluster for Systems Neurology (SyNergy), Munich, Germany; ^5^Institute of Clinical Neuroimmunology, University Hospital, LMU Munich, Munich, Germany; ^6^Data Integration for Future Medicine (DIFUTURE) Consortium, LMU Munich, Munich, Germany

**Keywords:** phenylketonuria (PKU), glial fibrillary acidic protein (GFAP), neurofilament light chain (NFL), Simoa assay, biomarkers brain alterations, optical coherence tomography, glial alterations, neuroaxonal damage

## Abstract

To pave the way for healthy aging in early treated phenylketonuria (ETPKU) patients, a better understanding of the neurological course in this population is needed, requiring easy accessible biomarkers to monitor neurological disease progression in large cohorts. The objective of this pilot study was to investigate the potential of glial fibrillary acidic protein (GFAP) and neurofilament light chain (NfL) as blood biomarkers to indicate changes of the central nervous system in ETPKU. In this single-center cross-sectional study, GFAP and NfL concentrations in serum were quantified using the Simoa^®^ multiplex technology in 56 ETPKU patients aged 6–36 years and 16 age matched healthy controls. Correlation analysis and hierarchical linear regression analysis were performed to investigate an association with disease-related biochemical parameters and retinal layers assessed by optical coherence tomography. ETPKU patients did not show significantly higher GFAP concentrations (mean 73 pg/ml) compared to healthy controls (mean 60 pg/ml*, p* = 0.140). However, individual pediatric and adult ETPKU patients had GFAP concentrations above the healthy control range. In addition, there was a significant association of GFAP concentrations with current plasma tyrosine concentrations (r = −0.482, *p* = 0.036), a biochemical marker in phenylketonuria, and the retinal inner nuclear layer volume (r = 0.451, *p* = 0.04). There was no evidence of NfL alterations in our ETPKU cohort. These pilot results encourage multicenter longitudinal studies to further investigate serum GFAP as a complementary tool to better understand and monitor neurological disease progression in ETPKU. Follow-up investigations on aging ETPKU patients are required to elucidate the potential of serum NfL as biomarker.

## Introduction

Phenylketonuria (PKU; OMIM #261600) is an autosomal recessive inborn error of phenylalanine metabolism. Reduced activity of the enzyme phenylalanine hydroxylase leads to impaired degradation of the amino acid phenylalanine (Phe) to tyrosine (Tyr), resulting in increased Phe levels and low Tyr levels in blood and brain, and subsequently reduced biosynthesis of biogenic amine neurotransmitters (1). If untreated, PKU leads to severe brain damage with intellectual disability, seizures, and spasticity (2).

The severe neurological phenotype of PKU has been prevented by the introduction of newborn screening enabling an early initiation of dietary therapy (3, 4) and/or cofactor treatment with BH_4_ (Kuvan^®^ sapropterin dihydrochloride) (early treated phenylketonuria, ETPKU) (5–7). However, ETPKU patients seem not to be free of sequelae (8). Several studies found decreased mean neurocognitive function levels, neuropsychiatric symptoms and imaging alterations such as white matter abnormalities and cerebral atrophy even in early and continuously treated patients (8–21). Furthermore, there are concerns that ETPKU patients may be more susceptible to develop classical neurodegenerative diseases (19, 20, 22), given the suggested chronic effects of Phe toxicity and disturbed Phe metabolism on the central nervous system (22–25). Eventually, the chronic effects of PKU on the brain of patients treated early and continuously remain elusive (19, 26, 27), not least because the first PKU patients who benefited from early treatment are only now reaching the sixth decade of life. These considerations have stimulated the current debate on treatment targets for ETPKU from the perspectives of overtreatment with medical, psychological, social, and economic consequences (28, 29) and undertreatment with possibly adverse outcome (27, 30).

To pave the way for healthy aging in ETPKU patients, we need a better understanding of the neurological course in this disease population, including the onset of age-related neuronal degeneration compared to healthy individuals and the extent to which the metabolic control throughout the patient's lifespan contributes to the neurological outcome (19). This requires easily accessible biomarkers to monitor neurological disease progression in large cohorts.

To date, neurodegeneration in ETPKU has been studied primarily by magnetic resonance imaging (MRI) (9–16, 20). However, MRI is a laborious and cost-intensive technology. As a result, larger multicenter longitudinal studies are limited in practicability.

Only recently, evidence for glial alterations and neuroaxonal degeneration was found in ETPKU patients using optical coherence tomography (OCT) (31–35). The hypothesis of retinal glial alterations and neuroaxonal damage associated with brain lesions in ETPKU (35) was strengthened by the finding of increased S-100B protein concentration in serum (36) and t-tau and p-tau in the cerebrospinal fluid of ETPKU patients (20).

Exploring glial and neuroaxonal damage, the brain-specific blood biomarkers glial fibrillary acidic protein (GFAP) and neurofilament light chain (NfL) come into focus. Elevated levels of GFAP, a cytoskeletal component of astrocytes (37–39), are associated with astrocyte activation or injury (40). NfL is a neuronal cytoskeletal component, and elevated levels of NfL are linked to neuroaxonal damage (37). With the development of an ultrasensitive single molecule array (Simoa^®^), GFAP and NfL have shown to be promising blood biomarkers for astrocytic response and neuroaxonal injury within the central nervous system in various neuroinflammatory and neurodegenerative disorders (37, 41). As GFAP and NfL are indicative of ongoing neuroinflammatory and neurodegenerative disease processes, they are of great use in detecting earliest neuropathological changes and provide information about neuropathological progression (42–46).

GFAP and NfL concentrations in blood have not yet been investigated in patients with ETPKU. The primary objective of the present study was to investigate GFAP and NfL concentrations in the serum (sGFAP, sNfL) of ETPKU patients compared with age-matched healthy controls. The secondary objectives were to determine whether the sGFAP and sNfL concentrations of ETPKU patients associate with disease-related biochemical parameters or OCT parameters.

## Materials and methods

### Study population

All patients who had been diagnosed with PKU by neonatal screening, and who were under regular care including routine blood sampling at the metabolic department of the Ludwig-Maximilians-University hospital in Munich, Germany were invited to participate in this study. Inclusion criteria were confirmed ETPKU and age 6 years and older. Exclusion criteria were chosen based on current knowledge of confounding effects of comorbidities (47, 48): (i) history of cardiovascular or cerebrovascular disease, cardiovascular risk factors (hypertension, poor glycemic control, smoking) and renal dysfunction, (ii) history of traumatic brain injury or any peripheral or central nervous disease unrelated to PKU, (iii) current pregnancy, and (iv) interfering medication or medical procedures. Exclusion criteria were identified based on the patient's medical history.

Ninty eligible patients were prospectively identified and approached about the possibility of study participation. 61 patients decided to participate, and of these 56 patients met the inclusion criteria and were prospectively included in the study between November 2020 and July 2021 (see Table 1). Five patients could not be included due to pregnancy.

**Table 1:** Demographic data, disease-related information, and OCT measures.

	**Pediatric ETPKU**		**Adult ETPKU**	
	**(*****N =*** **34)**		**(*****N =*** **22)**	
Age in years (range)	11 (7–17)		25 (18–36)	
Sex female *N* (%)	15 (44)		15 (68)	
**Comorbidities**				
Life-time depression *N* (%)			1 (2)	
Attention deficit/hyperactivity disorder *N* (%)	1 (3)			
Developmental delay *N* (%)	1 (3)		
Reading*/*spelling weakness *N* (%)	4 (12)		
Dyscalculia *N* (%)	1 (3)		
**BH**_**4**_**-responders** ***N*** **(%)**	20 (59)		14 (64)	
**Blood Phe [μmol/l]**	mean	SD	mean	SD
Childhood (0–10 years)				
IDC	234	52	283	103
Average of yearly SD	133	51	169	46
Adolescence (11–16 years)				
IDC	490	238	480	185
Average of yearly SD	146	68	145	51
Adulthood (17 years +)				
IDC	459	n.a.	599	270
Average of yearly SD	4.0	n.a.	135	48
Lifetime				
IDC	267	83	424	168
Mean Phe	269	82	417	141
Average of yearly SD	133	51	151	43
SD Phe	198	97	259	92
Current Phe	445	308	705	456
Past year				
Mean Phe	386	217	617	321
SD Phe	102	45	95	86
**Blood Tyr [μmol/l]**	51.6	22.9	42.8	14.6
**OCT measures**				
pRNFL [μm]	104	9.7	99	6.3
GCIPL [mm^3^]	0.60	0.05	0.59	0.04
INL [mm^3^]	0.26	0.03	0.24	0.02

IDC, average of yearly median phenylalanine levels; Phe, phenylalanine; Tyr, tyrosine; SD, standard deviation; ETPKU, early treated phenylketonuria; OCT, optical coherence tomography; pRNFL, peripapillary retinal nerve fiber layer thickness; GCIPL, combined ganglion cell and inner plexiform layer; INL, inner nuclear layer.

Eight pediatric (mean age 15 years, range 13-17, female 50%) and 8 adult (mean age 28.5 years, range 22–36, female 50%) healthy controls (HC) matched for age of the patients were also included in the study.

The study was performed in accordance with the Helsinki II Declaration and approved by the ethics committee of the Ludwig-Maximilians-University of Munich, Medical Faculty (project no 19-0453 and 20-997). All participants and/or their legal representatives gave written informed consent.

### Clinical data

Disease-related parameters were obtained from the patients' records.

First, disease type (BH_4_ responsive or non-responsive) was collected.

Second, indices for Phe control were determined. For the ETPKU patients, comprehensive Phe monitoring data were available. Limited data were available only for patients who were treated at other metabolic centers in childhood (*N* = 1), or had poor adherence in adolescence (*N* = 2) or adulthood (*N* = 1). Indices of Phe control based on dried blood spot measurement were calculated as described before (16, 35, 49, 50). We averaged Phe control in the following age bands: childhood 0–10 years of age, adolescence 11–16 years of age, adulthood 17 years of age to present, and lifetime. For each age band we considered the two measures Phe average and Phe variation (49). The Phe average was calculated by averaging the yearly median Phe levels (IDC). The Phe variation was calculated by averaging the standard deviation (SD) for each year (49). We furthermore calculated the mean (mean Phe) and SD (SD Phe) of all available Phe levels for each patient (16). Furthermore, we considered the mean and SD of all available Phe levels the year prior serum sampling, which is referred to hereafter as the mean/SD Phe of the past year, and the current plasma Phe concentration determined at the time of serum sampling for GFAP and NfL measurement.

Third, current plasma Tyr concentrations determined at the time of serum sampling were collected.

### Biomarker measurement

Serum samples were collected through venipuncture. Samples were allowed to clot at room temperature, subsequently centrifuged at 1,800 g for 10 min, and stored at −80°C in polypropylene tubes. Samples were thawed at room temperature and centrifuged at 10,000 g for 10 min. GFAP and NfL concentrations of ETPKU patients and pediatric HC were quantified with the Simoa^®^ Human Neurology 2-Plex B Kit (Quanterix) on a HD-1 analyzer^TM^ (Quanterix) according to manufacturer's instructions. GFAP and NfL concentrations in adult HC were quantified with the Simoa^®^ Human Neurology 4-Plex B Kit (Quanterix) on a HD-X analyzer^TM^ (Quanterix). All samples were measured in duplicates by aspirating two aliquots from a single well. The mean concentration of the two measurements was used for the analysis. In one patient, only one measurement could be obtained for each protein of interest. Given the low intra-sample variation between duplicate readings for the remaining samples in NfL and GFAP, these values were included in the analysis. The intra-assay coefficient of variation (CV) was calculated between duplicate readings for each protein. The mean CVs was 6.1% for GFAP and 7.1% for NfL. Samples with CVs above 20% were excluded from further analysis (GFAP *n* = 1, NfL *n* = 2). The inter-assay CV of two quality control samples over the two runs was on average 5.6% CV for GFAP and 1.9% CV for NfL.

### Spectral-domain optical coherence tomography (OCT)

OCT examination was performed using a SD-OCT (Spectralis, Heidelberg Engineering, Heidelberg, Germany) with automatic real time (ART) function for image averaging as described before (35). Data are reported for peripapillary retinal nerve fiber layer thickness (pRNFL) to assess axonal degeneration, volume of combined ganglion cell and inner plexiform layer (GCIPL = GCL + IPL) as marker for neuronal degeneration, and for inner nuclear layer (INL). Macular layers were calculated for a 3 mm diameter cylinder around the fovea from a macular volume scan (20° x 20°, 25 vertical B-scans, ART ≤ 49). The pRNFL was measured with activated eye tracker using 3.4 mm ring scans around the optic nerve (12°, 1536 A-scans, ART ≤100). Segmentation of all layers was performed semi-automatically using software provided by the OCT manufacturer (Eye Explorer 1.9.10.0 with viewing module 6.3.4.0, Heidelberg Engineering, Heidelberg, Germany). All scans were checked for sufficient quality and segmentation errors and corrected, if necessary. OCT data are reported according to the APOSTEL (2.0) and OSCAR-Ib recommendations (51–53). Both eyes of each subject were included in subsequent analysis as statistically dependent duplicates.

### Statistical analyses

Statistical analyses were performed using SPSS Statistics 26 (IBM) by the authors (ASL-H).

Comparison of demographic data between the patient and control group was analyzed by Chi-Square test. Spearman correlation analysis and curve fitting regression analysis were performed to analyze associations of sGFAP and sNfL concentrations with age.

To compare ETPKU patients with HC, the Mann-Whitney-U test was applied.

Subsequent analyses evaluating an association between biomarkers and disease-related parameters or OCT parameters were performed following natural log transformation of biomarker concentrations (47). Normal distribution of transformed biomarkers was tested with distributional plots and the Shapiro–Wilk test.

At first, partial correlation analysis controlling for age was applied to investigate an association of disease-related parameters and biomarkers in ETPKU.

Additionally, hierarchical linear regression analysis was computed to examine the contribution of disease-related parameters to serum biomarkers in ETPKU patients. The independent variables were entered into the equation in the step-wise manner as follows: BH_4_ responsiveness, Phe indices (IDC, average of yearly SD, current Phe concentration, mean/SD Phe of the past year), and current Tyr concentration. Age as potential confounder was also added to the model. An autocorrelation scatter plot and the Durbin-Watson (DW) statistic were applied to account for autocorrelation. No autocorrelation was assumed for a random distribution in the scatter plot and a DW value between 1.5 and 2.5. The variance inflation factor (VIF) was calculated to test for multicollinearity. Critical levels of multicollinearity were assumed if VIF was greater than 5.

For two reasons, correlation and regression analyses were performed only for the group of ETPKU patients 18–36 years of age (*N* = 22): (i) as expected from the literature (54), sGFAP and sNfL concentrations were not associated with age in this cohort, (ii) documentation of Phe levels from childhood, adolescence, and adulthood was available.

For all analyses, *p* ≤ 0.05 were considered significant.

## Results

### Patients' characteristics and disease features

Table 1 shows demographic data, disease-related parameters and OCT measures.

59% of the pediatric and 64% of the adult ETPKU patients were BH_4_-responsive. On average, the ETPKU patients showed a good Phe control in childhood, adolescence, and adulthood (27). Variability in Phe was largely consistent across all age ranges.

Current Phe (705 μmol/l) and mean Phe of the past year (617 μmol/l) in the adult cohort were slightly above the target range recommended in Europe (27).

The average Tyr concentrations in plasma were within the reference range (34–112 μmol/l) in pediatric (51.6 μmol/l) and adult ETPKU patients (42.8 μmol/l). Five pediatric and eight adult ETPKU patients had current Tyr concentrations in plasma below the reference range (mean ± SD 28.4 ± 2.9 and 30.4 ± 2.0).

All ETPKU patients attended regular school, were socially integrated and active in their lives, as confirmed by partners or parents. 14% of the ETPKU patients had been diagnosed with comorbidities such as depression, attention deficit hyperactivity disorder, developmental delay, reading*/*spelling weakness, or dyscalculia, that were still present at the time of serum sampling (Table 1).

The patients examined in this study were part of a previously published cohort of PKU patients studied with OCT (35). The pRNFL thickness and GCIPL and INL volumes of the ETPKU patients in this study are shown in Table 1.

### sGFAP levels in ETPKU

**Figure 1:**
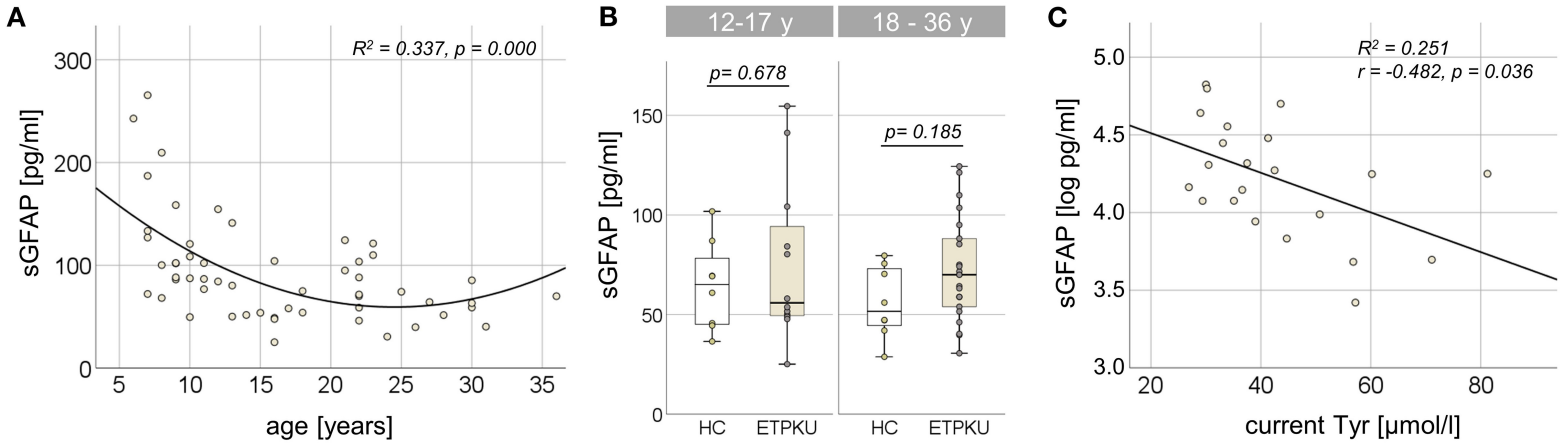
Serum glial fibrillary acidic protein (sGFAP) in early treated phenylketonurie (ETPKU) patients. **(A)** Association of sGFAP concentrations and age. The quadratic relationship between sGFAP concentrations and age is depicted by the continuous line. **(B)** sGFAP concentrations in ETPKU 12-17 (*N* = 12) and 18-36 (*N* = 22) years of age compared to healthy controls (HC; *N* = 8 / 8). **(C)** Association of natural log-transformed sGFAP concentrations in adult ETPKU (*N* = 22) and current concentrations of tyrosine (Tyr) in plasma. The relationship of the variables was linear, as depicted by the continuous line.

In the entire ETPKU cohort (*N* = 56, 6–36 years of age), there was a quadratic relationship between sGFAP concentrations and age (R^2^ = 0.337, *p* = 0.000) with a negative association of sGFAP concentrations and age below an age of 12 years (Figure 1A). Maximum sGFAP concentrations were 171 ± 68 pg/ml at ≤7 years of age, with a steep decline in sGFAP concentrations by 9 years of age. No significant correlation of sGFAP concentrations and age was observed in ETPKU with 12–36 years of age (*N* = 34, mean age 21 years, r = −0.148, *p* = 0.403). Correlation analysis of the age matched HC cohort also showed no association of sGFAP with age in this age range (*N* = 16, mean age 22 years, r = −0.068, *p* = 0.717).

In comparison to the HC cohort, ETPKU patients in this age range did not show significantly different sGFAP concentrations (ETPKU; mean 73 pg/ml, SD 31 pg/ml, CI 63–84 pg/ml, median 67 pg/ml, IQR 51–89 pg/ml vs. HC; mean 60 pg/ml, SD 20 pg/ml, CI 49–71 pg/ml, median 58 pg/ml, IQR 45–74 pg/ml, *p* = 0.140). There were also no significant differences of sGFAP concentrations in ETPKU patients compared to HC when the pediatric (ETPKU; *N* = 12 and HC; *N* = 8) and adult (ETPKU; N = 22 and HC; N = 8) cohorts were considered separately [12–17 years; ETPKU mean 75 pg/ml, SD 40 pg/ml, CI 50–100 pg/ml, median 56 pg/ml, IQR 49–99 pg/ml vs. HC mean 64 pg/ml, SD 22 pg/ml, CI 46–83 pg/ml, median 65 pg/ml, IQR 45−83 pg/ml, *p* = 0.678, 18–36 years; ETPKU mean 73 pg/ml, SD 26 pg/ml, CI 61–84 pg/ml, median 70 pg/ml, IQR 53–90 pg/ml vs. HC mean 56 pg/ml, SD 18 pg/ml, CI 41–71 pg/ml, median 52 pg/ml, IQR 43–74 pg/ml, *p* = 0.185] (Figure 1B). However, two pediatric and seven adult ETPKU patients showed sGFAP concentrations above the HC range (Figure 1B).

### Association between sGFAP and disease-related parameters

As shown in Figure 1C, log-transformed sGFAP concentrations significantly correlated with current Tyr concentrations (r = −0.482, *p* = 0.036). No significant correlation of log-transformed sGFAP concentrations was observed with any of the Phe indices (IDC, average of yearly SD, current Phe concentration, mean/SD Phe of the past year) (for all *p* > 0.05) (Table 2).

**Table 2:** Partial correlation analysis of log-transformed sGFAP and sNfL concentrations in adult ETPKU patients with disease-related parameters.

**Disease-related parameters**	**log (sGFAP)**		**log (sNfL)**	
	**r**	***p***	**r**	***p***
IDC 0–10 yrs	0.100	0.684	0.083	0.735
IDC 11–16 yrs	−0.021	0.932	0.143	0.559
IDC 17 yrs +	0.011	0.966	0.202	0.408
Past year mean Phe	0.120	0.624	0.406	0.085
Current Phe	0.025	0.920	0.344	0.150
Average yearly SD 0–10 yrs	0.027	0.912	0.236	0.330
Average yearly SD 11–16 yrs	0.171	0.485	0.439	0.060
Average yearly SD 17 yrs +	0.238	0.328	0.221	0.362
Past year SD Phe	−0.159	0.515	0.014	0.954
Current Tyr	−0.482	0.036^*^	−0.029	0.907

log(sGFAP), log-transformed serum glial fibrillary acidic protein; log(sNfL), log-transformed serum neurofilament light chain; IDC, average of yearly median phenylalanine levels; Phe, phenylalanine; Tyr, tyrosine; SD, standard deviation; r, correlation coefficient.

* p ≤ 0.05. r- and p-values were calculated using partial correlation analysis controlling for age.

In the hierarchical linear regression model used to analyze a contribution of disease-related parameters on log-transformed sGFAP concentrations (adjusted r^2^ = 0.210), current Tyr concentration was the only significant predictor (β = −0.501, *p* = 0.024) (Table 3). There was no indication for auto-correlation (Durbin-Watson 2.1) or multicollinearity (VIF 1.0). All other independent variables (BH_4_-responsiveness, Phe indices, and age) did not reveal a significant contribution and were thus excluded from the model (Table 3).

**Table 3:** Hierarchical linear regression analysis to predict a contribution of disease-related parameters on log-transformed sGFAP or sNfL concentrations in adult ETPKU patients.

**Disease-related parameters**	**log(sGFAP)**			**log(sNfL)**		
	**β**	***p***	**VIF**	**β**	***p***	**VIF**
Age	−0.153	0.476	1.028	0.610	0.004^*^	1.000
BH_4_-responsiveness	0.228	0.295	1.080	0.183	0.343	1.010
IDC 0–10 yrs	−0.030	0.887	1.001	0.078	0.735	1.408
IDC 11–16 yrs	−0.062	0.772	1.006	0.121	0.559	1.138
IDC 17 yrs +	−0.184	0.409	1.114	0.160	0.408	1.004
Past years mean Phe	0.085	0.689	1.007	0.322	0.085	1.002
Current Phe	−0.010	0.961	1.011	0.273	0.150	1.007
Average yearly SD 0–10 yrs	−0.136	0.536	1.069	0.189	0.330	1.013
Average yearly SD 11–16 yrs	0.099	0.643	1.006	0.350	0.060	1.016
Average yearly SD 17 yrs +	0.167	0.433	1.017	0.175	0.362	1.000
Past year SD Phe	−0.157	0.461	1.014	0.012	0.954	1.059
Current Tyr	−0.501	0.024^*^	1.000	−0.023	0.907	1.028

log(sGFAP), log-transformed serum glial fibrillary acidic protein; log(sNfL), log-transformed serum neurofilament light chain; BH4, sapropterin dihydrochloride; IDC, average of yearly median phenylalanine levels; Phe, phenylalanine; Tyr, tyrosine; SD, standard deviation; β, beta coefficient.

* p ≤ 0.05. VIF; variance inflation factor. β-, p-, and VIF-values were calculated using hierarchical linear regression analysis.

### sNfL levels in ETPKU

**Figure 2:**
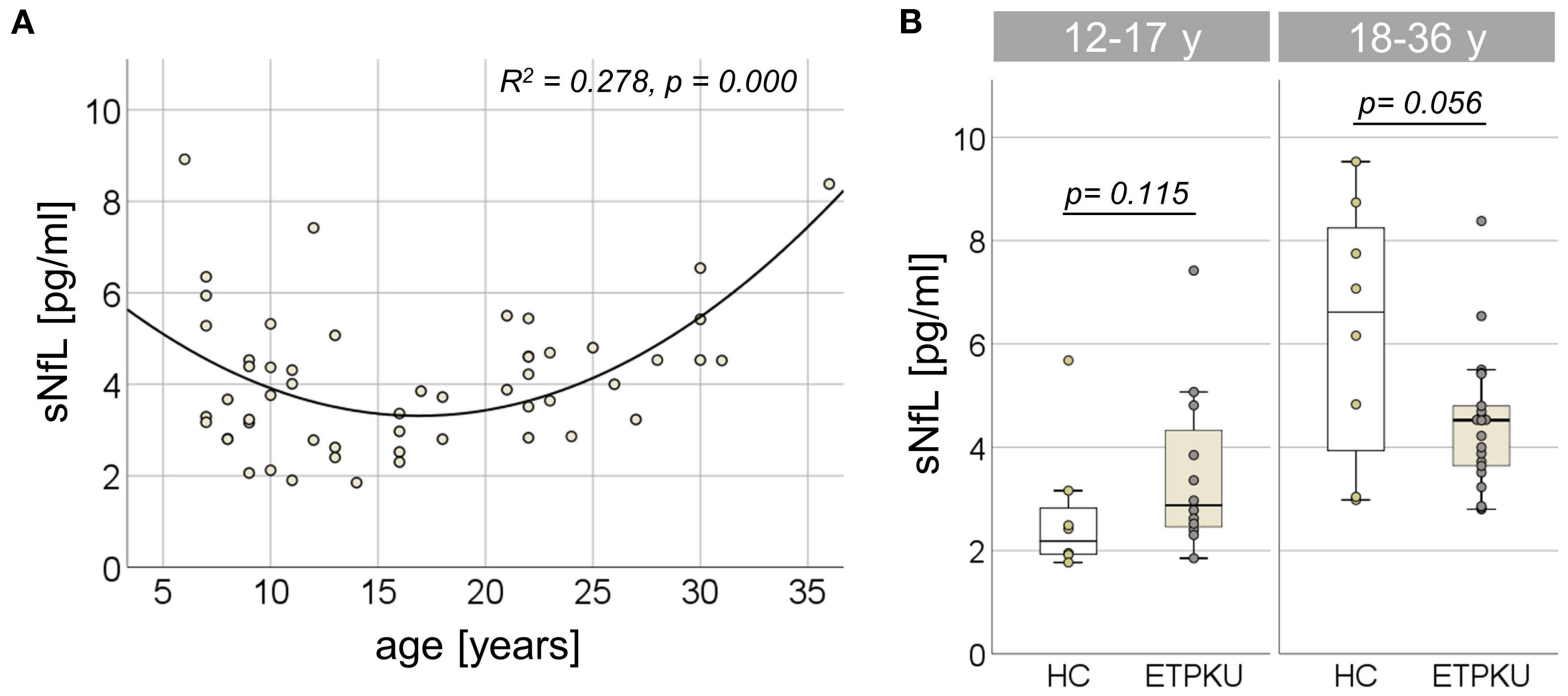
Serum neurofilament light chain (sNfL) in early treated phenylketonurie (ETPKU) patients. **(A)** Association of sNfL concentrations and age. The quadratic relationship between sNfL concentrations and age is depicted by the continuous line. **(B)** sNfL concentrations in ETPKU 12-17 (*N* = 12) and 18-36 (*N* = 18) years of age compared to healthy controls (HC; *N* = 8 / 8).

In the entire ETPKU cohort (*N* = 56, 6–36 years of age), there was a quadratic relationship between sNfL concentrations and age (R^2^ = 0.278, *p* = 0.000) with a negative association of sNfL concentrations and age below an age of 12 years and a positive association above 12 years of age (Figure 2A). The maximum sNfL concentration was 8.9 pg/ml at 6 years of age. No significant correlation of sNfL concentrations and age was observed in ETPKU patients with 12-17 years of age (*N* = 12, mean age 14 years, r = −0.147, *p* = 648) or 18–36 years of age (*N* = 22, mean age 25 years, r = 0.369, *p* = 0.091). This was also true for the age matched HC cohort (12–17 years; *N* = 8, mean age 15 years, r = −0.061, *p* = 885, 18–36 years; *N* = 8, mean age 29 years, r = 0,675, *p* = 0.066).

In both age groups, there was no significant difference of sNfL concentrations in ETPKU patients compared to HC [12–17 years; ETPKU mean 3.4 pg/ml, SD 1.6 pg/ml, CI 2.3–4.5 pg/ml, median 2.9 pg/ml, IQR 2, 4–4,6 pg/ml vs. HC mean 2.7 pg/ml, SD 1.3 pg/ml, CI 1.6–3.8 pg/ml, median 2.2 pg/ml, IQR 1.9–3.0 pg/ml, *p* = 0.115, 18–36 years; ETPKU mean 4.5 pg/ml, SD 1.3 pg/ml, CI 3.8–5.0 pg/ml, median 4.5 pg/ml, IQR 3.6–5.0 pg/ml vs. HC mean 6.2 pg/ml, SD 2.5 pg/ml, CI 4.2–8.3 pg/ml, median 6.6 pg/ml, IQR 3.5–8.5 pg/ml, *p* = 0.056 (Figure 2B)].

### Association between sNfL levels and disease-related parameters

Partial correlation analysis adjusted for age revealed no association of log-transformed sNfL concentrations with any of the Phe indices (IDCs, average of yearly SDs, current Phe concentration, mean Phe of the past year) or current Tyr concentration (for all *p* > 0.05) (Table 2).

In the hierarchical linear regression model used to analyze a contribution of disease features on log-transformed sNfL concentrations (adjusted r^2^ = 0.338), the age was the only predictor (β = 0.610, *p* = 0.004). There was no indication for auto-correlation (Durbin-Watson 2.15) or multicollinearity (VIF 1.0). All other independent variables (BH_4_-responsiveness, all Phe indices, and current Tyr concentration) did not reveal a significant contribution and were thus excluded from the model (Table 3).

### Correlation of serum biomarker levels and OCT measures

Partial correlation analysis adjusted for age of log-transformed sGFAP concentrations with the optical measures pRNFL, GCIPL and INL (Table 4) showed a significant association with the INL (r = 0.451, *p* = 0.040). No correlation of sNfL with any of the OCT measures was found (for all *p* > 0.05) (Table 4).

**Table 4:** Partial correlation analysis of log-transformed sGFAP and sNfL concentrations in adult ETPKU patients with OCT measures.

**OCT measures**	**log(sGFAP)**		**log(sNfL)**	
	**r**	***p***	**r**	***p***
pRNFL	−0.164	0.477	−0.031	0.893
GCIPL	0.036	0.877	0.076	0.742
INL	0.451	0.040^*^	0.328	0.147

OCT, optical coherence tomography; log(sGFAP), log-transformed serum glial fibrillary acidic protein; log(sNfL), log-transformed serum neurofilament light chain; pRNFL, peripapillary retinal nerve fiber layer thickness; GCIPL, combined ganglion cell and inner plexiform layer; INL, inner nuclear layer; r, correlation coefficient.

* p ≤ 0.05. r- and p-values were calculated using partial correlation analysis controlling for age.

## Discussion

This study was performed to investigate the potential of GFAP and NfL as blood biomarkers for astrocytic response and neuroaxonal injury in ETPKU patients. Our data did not show significantly different sGFAP levels in ETPKU compared to HC. However, it must be mentioned that individual pediatric and adult ETPKU patients showed clearly higher sGFAP levels than the range of HC. Analyzing a possible association between the biomarker concentrations, disease-related biochemical parameters, and OCT parameters, we found a correlation of sGFAP with current plasma Tyr concentrations and with the retinal inner nuclear layer volume. There was no evidence of sNfL alterations in our ETPKU cohort.

### Evidence for sGFAP elevation in ETPKU

The negative association of sGFAP concentrations with age in our pediatric ETPKU cohort below 12 years of age is in line with previously published data in HC (55). The age dependent sGFAP concentrations were within similar ranges in our ETPKU cohort compared to published HC (55).

Also in adolescent (12–17 years) and adult patients (18–36 years), our data did not indicate significantly elevated levels of sGFAP in ETPKU. Larger, multicenter studies and follow-up investigations are needed to clarify the observation that sGFAP concentrations of individual ETPKU patients were above the HC range (39, 41, 56).

None of the patients of our ETPKU cohort had symptoms indicative of advanced neurodegenerative disease. However, it has recently been shown for Alzheimer‘s disease that sGFAP is a promising biomarker to identify patients at-risk (42, 57). Therefore, one could speculate that also in ETPKU, sGFAP might be a biomarker to identify patients at-risk for neurodegenerative disease, a hypothesis that needs further investigation in prospective studies.

In accordance with our observation of sGFAP concentrations above the HC range in individual ETPKU patients, a previous study in the *Pah*^enu2^ mouse, an animal model for PKU, demonstrated that oligodendrocytes expressed GFAP filaments in response to Phe loading (58). It is important to note that the *Pah*^enu2^ mice in this study were exposed to increasing concentrations of Phe, whereas all patients included in our study were following therapy and therefore did not have their (high) baseline Phe levels at the time of sGFAP sampling. This might be the reason, why we found no correlation between sGFAP concentrations and any of the calculated Phe indices, including the current Phe level. Beyond this, there is evidence that the processes in the brain of PKU patients are due to secondary disturbances rather than solely to neurotoxic effects of high Phe levels (59). This was also suggested by a recent study using organic hippocampal slice cultures as a model to study glial integrity in PKU, in which unchanged GFAP expression was observed upon exposure to high vs. physiological concentrations of Phe (60).

Interestingly, sGFAP concentrations in adult ETPKU patients correlated negatively with current plasma Tyr concentrations, and current Tyr concentrations were a significant predictor of sGFAP concentrations in hierarchical linear regression analysis. The negative association of current plasma Tyr and sGFAP concentrations may be explained by the following considerations: Reduced Tyr concentrations in plasma have been considered to be an indicator for low cerebral Tyr and dopamine concentrations (31, 61), and dopamine deficits have been suggested to contribute to brain damage in adult PKU patients (22). As astrocytes have been demonstrated to respond to dopamine released at synaptic sites (62), one might suppose that altered dopamine concentrations have a negative effect on astrocyte stimulation. For their part, astrocytes (and other astroglial cells) respond to neuropertubative conditions with cell hypertrophy and GFAP expression (40).

In addition, our analyses revealed a correlation of sGFAP concentrations with the retinal INL volume. In the retinal INL, the Mueller cells, astroglial cells of the retina, are located. Like other astroglial cells, hypertrophy and GFAP expression have also been demonstrated for Mueller cells as a reaction to tissue stress (63–66). Considering this, one may hypothesize that neuropertubative conditions in ETPKU patients, such as biogenic amine neurotransmitters depletion and oxidative stress (22, 24), lead to a general astroglial response (in astrocytes and Mueller cells) reflected by elevated sGFAP concentrations (mainly in astrocytes) and higher INL volume (in Mueller cells).

### sNfL is not altered in pediatric and young adult ETPKU

No alterations of sNfL concentrations in ETPKU patients were found in this study. The negativ association of sNfL concentrations with age in children below 12 years of age and the positive association in adults were consistent with data from HC previously described (55, 67–69). The sNfL concentrations in our ETPKU cohort were not elevated compared to our age-matched healthy cohort, and were within previously published percentiles (54, 55, 67–71), assuming that data collected with other testing methods are comparable (37, 72).

In contrast to sGFAP that is promising in identifying patients at-risk for neurodegenerative disease such as Alzheimer‘s disease, sNfL as a biomarker detecting axonopathy is considered to be more helpful in discriminating later stages (42, 57). On this basis, one could speculate that the normal sNfL levels in our ETPKU cohort, also in those patients with sGFAP concentrations above the HC range, are due to the absence of advanced, clinically relevant astrogliosis. In addition, the ETPKU cohort studied is still quite young—like all PKU patients who benefited from early and continuous treatment.

With regards to the concern that ETPKU patients may be more susceptible to develop classical neurodegenerative disease (19, 20, 22), longitudinal studies with repeated sNfL investigation in the future elderly PKU patients treated early and continuously may contribute to understand whether there is a potential value of sNfL as a biomarker of neuroaxonal damage in ETPKU.

### Limitations of the study

ETPKU is a rare disease and various disease-related parameters might have an impact on neuropathological processes. A potential bias could be caused by study-specific characteristics (e.g., phenotype, metabolic control) as well as the small sample size. The latter might be one reason why the results of the sGFAP evaluation did not reach the level of significance.

A possible influence on biomarker concentrations could also result from unrecognized comorbidities. We aimed to minimize the risk of influence by choosing the exclusion criteria, and the bias would have affected both the ETPKU and HC cohort. Nevertheless, larger studies are needed to minimize the potential bias. Both, sGFAP and sNfL measurements are promising for multicenter approaches enabling future cross-sectional and longitudinal studies (41, 73).

Another limitation of this study is that biomarker concentrations were quantified in adult HC with a different assay (Human Neurology 4-Plex B) than in the ETPKU and pediatric HC cohorts (Human Neurology 2-Plex B). However, according to the manufactures data sheet (Quanterix Corporation, Inc. Simoa^TM^), comparable values are expected for both assays. When comparing the two assays, one would expect lower concentrations for the Human Neurology 2-Plex B Kit if there were any differences at all.

## Conclusion

These pilot results motivate multicenter longitudinal studies to further investigate blood-based biomarkers such as GFAP and NfL as a complementary tool to better understand and monitor neurological disease progression in ETPKU for the following three reasons. First, our data did not show significantly elevated sGFAP levels in ETPKU. Nevertheless, based on the sGFAP levels that were above the HC range in individual ETPKU patients, active astrogliosis can be suspected in these patients. Second, the negative association with plasma Tyr concentrations and the correlation with retinal INL volume may suggest a common pathomechanism based on disturbed dopamine metabolism and tissue stress, a question necessitating also further basic research. And third, in our rather young ETPKU cohort, sNfL levels were not increased as a marker of neuroaxonal damage compared with HC, but follow-up studies in aging ETPKU patients will elucidate the potential of sNfL as biomarker.

## Data availability statement

The original contributions presented in the study are included in the article, further inquiries can be directed to the corresponding authors.

## Ethics statement

The studies involving human participants were reviewed and approved by Ethics Committee of the Ludwig-Maximilians-University of Munich, Medical Faculty. Written informed consent to participate in this study was provided by the participants' legal guardian/next of kin.

## Author contributions

AL-H designed and conceptualized study, major role in the acquisition of data, statistically analyzed and interpreted the data, and drafted the manuscript. JH designed and conceptualized study, interpreted the data, and drafted the manuscript. EM design and conceptualized study and drafted the manuscript. BN acquisition of data. SK and JL acquisition and interpretation of data. All authors contributed to the final version of the manuscript.

## Funding

This work was supported by a research grant from Nutritia Metabolics and by the Deutsche Forschungsgemeinschaft (DFG, German Research Foundation) under Germany's Excellence Strategy within the framework of the Munich Cluster for Systems Neurology (EXC 2145 SyNergy – ID 390857198). The funders were not involved in the study design, collection, analysis, interpretation of data, the writing of this article, or the decision to submit it for publication.

## Conflict of interest

Author AL-H has received travel reimbursement from BioMarin, Nutritia Metabolics, and Sobi and author fees from Thieme medical publishers. Authors JL and SK were funded by the Deutsche Forschungsgemeinschaft (DFG) under Germany's Excellence Strategy within the framework of the Munich Cluster for Systems Neurology (EXC 2145 SyNergy – ID 390857198). Author JL reports speaker fees from Bayer Vital, Biogen and Roche, consulting fees from Axon Neuroscience and Biogen, author fees from Thieme medical publishers and W. Kohlhammer GmbH medical publishers. In addition, he reports compensation for serving as chief medical officer for MODAG GmbH, is beneficiary of the phantom share program of MODAG GmbH and is inventor in a patent Pharmaceutical Composition and Methods of Use (EP 22 159 408.8) filed by MODAG GmbH, all activities outside the submitted work. Author JH is (partially) funded by the German Federal Ministry of Education and Research [Grant Numbers 01ZZ1603[A-D] and 01ZZ1804[A-H] (DIFUTURE)]. Author JH reports a grant for OCT research from the Friedrich-Baur-Stiftung, personal fees and non-financial support from Merck, Alexion, Novartis, Roche, Santhera, Biogen, Heidelberg Engineering, Sanofi Genzyme and non-financial support of the Guthy-Jackson Charitable Foundation, all outside the submitted work. Author EM has received a research grant from Nutritia Metabolic for this work and travel reimbursement from Sobi and Dr. Schär and was paid for advisory services from Sobi, APR and Sanofi-Aventis. The remaining authors declare that the research was conducted in the absence of any commercial or financial relationships that could be construed as a potential conflict of interest.

## Publisher's note

All claims expressed in this article are solely those of the authors and do not necessarily represent those of their affiliated organizations, or those of the publisher, the editors and the reviewers. Any product that may be evaluated in this article, or claim that may be made by its manufacturer, is not guaranteed or endorsed by the publisher.
